# Risky sexual behaviours and utilization of HIV testing services among the adolescent girls and young women aged between 15-24 years in Kibra Sub County, Nairobi County, Kenya

**DOI:** 10.12688/openreseurope.17609.1

**Published:** 2024-05-29

**Authors:** Onesmus Muti Mutie, Kenneth Ngure, Aggrey Gisiora Mokaya

**Affiliations:** 1 Department of Environmental Health and Disease Control, School of Public Health, Jomo Kenyatta University of Agriculture and Technology, Nairobi, Nairobi County, Kenya; 2 Center for Public Health Research, Kenya Medical Research Institute and Training Programmes Department, Kenya Medical Research Institute, Nairobi City, Kenya

**Keywords:** HIV Testing, Adolescent Girls, Young Women, Risky Sexual Behaviours, HIV Prevention

## Abstract

**Background:**

HIV remains a significant global health challenge, disproportionately affecting adolescent girls and young women (AGYW). HIV testing is crucial in controlling transmission and reducing its prevalence. Understanding risky sexual behaviours among AGYW is pivotal in aligning prevention interventions. Despite global prevention efforts, testing gaps persist among AGYW, linked to risky sexual behaviour (RSB). This study explores the association between these behaviours and HIV testing utilization among AGYW (aged 15–24) in Kibra Sub County, Nairobi.

**Methods:**

A cross-sectional study sampled 379 AGYW from three wards in Kibra Sub County in Nairobi County. To be an eligible participant, one must have been a resident for at least one year before the time of the study and aged between 15–24 years, employing standardized structured interviewer-administered questionnaires and statistical analyses. Results were analysed using Chi-square tests and logistic regression. Data was collected between June to July 2023.

**Results:**

Overall, HIV testing prevalence was 60.7% (n=230). Those aged 20–24 were 71.3% (n=164), with secondary education were 63.5% (n=146) and married 28.7% (66) were more likely to undergo testing. Participants engaging in risky behaviours such as lack of condom use (3.96 times more likely), experiencing gender-based violence (4.65 times more likely), or contracting STIs (2.85 times more likely) had higher odds of seeking HIV testing services.

**Conclusions:**

This study establishes a clear link between risky sexual behaviours and HIV testing among AGYW, with a 60.7% testing prevalence; however, gaps still exist. Efforts to enhance testing rates are vital. Interventions should align with acceptable methods, focusing on this affected community to ensure effective HIV care and prevention.

## Plain Language Summary

Localized insights into testing disparities: This study provides localized insights into HIV testing disparities within specific communities, shedding light on differences in testing rates among various wards. Understanding these community-specific variations is essential for tailoring targeted interventions and healthcare resources effectively.

Comprehensive analysis of sociodemographic factors: Unlike some previous studies, this research comprehensively examines a range of sociodemographic factors, including education, employment status, income, and marital status, to identify nuanced influences on HIV testing among Adolescent Girls and Young Women (AGYW). By analysing these multifaceted factors, this study enriches the understanding of the complex interplay between sociodemographic variables and HIV testing behaviour in this vulnerable population.

Behavioural contextualization of HIV testing: This study goes beyond numerical analysis by delving into the behavioural context surrounding HIV testing among AGYW. By exploring risky sexual behaviours in detail, such as transactional sex, alcohol-induced encounters, and STI diagnoses, this research offers a nuanced understanding of the psychosocial factors influencing HIV testing decisions. These insights are pivotal for developing targeted behavioural interventions that address the specific challenges faced by AGYW, thus contributing significantly to the design of more effective public health strategies.

## Introduction

HIV continues to be a major public health issue with over 38 million people acquiring HIV globally and about 3.4 million adolescent girls and young adults aged between 15–24 years living with HIV ^1^. HIV testing is the first step in HIV prevention of new transmissions and AIDS-related deaths, initiation on ART and improvement of quality of life among the Adolescent Girls and Young Women (AGYW) ^2^. Testing services have become increasingly available in Kenya with over 7,000 government health facilities offering the service for free and private health facilities with minimal charges ^3^. However, AGYW continue to be a uniquely vulnerable population that is exposed to risks that affect their health and well-being including risky sexual behaviours that expose them to HIV ^4^. The prevalence of HIV among AGYW in Kenya is two to three times that of adolescent boys and young men (ABYM) ^5^. Young people (15–24 years) contribute 41% of all new HIV diagnoses in the 15+ population in the country, with 70% of those new diagnoses in females ^3^. Further, UNAIDS estimates that every week, approximately 6200 AGYW aged between 15–24 years of age get newly transmitted with HIV in Kenya ^6^.

HIV transmission among the AGYW in Sub-Saharan Africa and other African Countries is mainly through the unprotected heterosexual route fuelled by risky sexual behaviours such as early sexual debut, low condom use, substance use, having multiple sexual partners, transactional sex, among others ^5^. In Nigeria for example, poverty, being orphan, dropping out of school, early child marriages and sexual violence among other inequalities are attributed to risks of unprotected sex among the AGYW ^7^.

A study done in Zimbabwe found out that AGYW who engage on sex for monetary gains are less likely to utilize HIV testing services compared to those who don’t do it for money, those who have multiple sexual partners are likely to test compared to those with only one and similarly, those who use condoms are less likely to test compared to those who don’t use ^8^. A similar study in Ethiopia among the AGYW, found that those who had multiple sexual partners, those who ever had been pregnant, started sexual intercourse early and had history of sexually transmitted infections were more likely to utilize HIV testing services due to the increased risk of HIV transmission ^9^. Additionally, studies across different parts of the globe have positively associated risky sexual behaviours such as age of respondents, early sexual debut, stigma, attitudes with predisposing AGYW to HIV and utilization of HIV testing services ^10– 12^.

Kenya has continually endeavoured to halt and reverse the spread of HIV transmission with remarkable achievements towards HIV prevention, and continues to implement various interventions geared towards achieving the UNAIDS 95-95-95 targets by 2030 ^3^. However, despite the efforts testing gaps exist for AGYW at 44% ^13^. HIV testing among AGYW in Ethiopia is 27% ^9^. Similar population-based survey in Malawi, Zambia and Zimbabwe showed that 46% of AGYW were aware of their HIV status ^14^. Several other studies in different countries have shown low HIV testing rates among AGYW such as Nigeria 12%, Uganda 26.5% and Rwanda 40% ^15^. A household study conducted in Viwandani and Korogocho slums in Nairobi showed that AGYW are less aware of their HIV status compared to those above 25 years ^16^.

Numerous investigations have focused on HIV among AGYW; however, a literature search failed to identify studies examining the nexus between risky sexual behaviours and the utilization of HIV testing services within this high-priority demographic. Consequently, this research aims to elucidate the prevalence of HIV testing and scrutinize the interplay among sociodemographic characteristics, risky sexual behaviours, and the utilization of HIV testing services among AGYW aged 15–24 in Kibra Sub County, Nairobi County, Kenya. The outcomes of this study stand poised to offer valuable insights for refining extant prevention strategies concerning differentiated HIV testing services. Moreover, the findings are anticipated to play a pivotal role in the formulation of targeted behavioural interventions tailored to address the specific challenges confronted by AGYW. Consequently, the study holds the potential to make significant contributions to the development of more efficacious public health strategies geared towards achieving HIV epidemic control among this vulnerable population by the year 2030.

## Methods

### Design

A community-based cross-sectional analytical study design, using quantitative techniques, was employed.

### Study site and population

Kibra is one of the 17 sub counties in Nairobi County, it is located southwest of Nairobi County approximately 5 kilometres from CBD and projected to have a population of 206,064 persons, 21.5% of this population constitutes the AYPs aged between 15–24 years. Majority of the residents are in informal settlements ^17^. The sample size of 379 adolescent girls and young women (AGYW) aged 15–24 was calculated using the Cochran formula ^18^. Employing a multistage sampling technique, three out of five Wards were randomly selected. Then random walk sampling method was used, starting from common meeting points (markets, hospitals, or schools) in selected wards. A local guide assisted in choosing a random route, with data collection continuing until the minimum sample size was reached. In households with multiple AGYW, random selection using yes/no indicators determined the participant. To be eligible participant must have been a resident for at least one year before time of study, consent/assent to participate and aged between 15–24 years.

### Dependent variable

The dependent variable of this study was utilization of HIV testing services among AGYW aged between 15–24 years in Kibra Sub County, which was measured by asking the question to the respondents whether have ever been tested for HIV and the outcome was binary (Yes/No).

### Independent variables

Independent variables for this study were grouped into two categories i.e. (sociodemographic characteristics and risky sexual behaviours) where Socio-demographic factors included (age, education level, employment status, average monthly income, marital status and religion). Risky sexual behaviours (ever had sex, age at first sexual debut, ever tested for HIV, had sex for cash, having multiple sexual partners, condom use in sexual encounters and if ever experienced gender-based violence).

### Inclusion criteria

The study included AGYW aged between 15–24 years who are residents of Kibra Sub County for at least one year.

### Exclusion criteria

The study excluded eligible AGYW 15–24 years not willing to consent or assent or whose parent or guardian declined to give consent and those absent from the selected households during data collection

### Sample size determination

Sample size was calculated using Cochran formula (1977)

### Sampling technique

The study used a four multistage sampling technique

### Pre testing of data collection tools

Research assistants were trained on the data collection tools then pretesting of the data collection questionnaire was done in 30 households where 30 interviewer administered questionnaires were administered to AGYW in Mathare Sub County. Mathare Sub County was chosen because the population there has similar characteristics like the one found in Kibra Sub County, this enabled the researcher to make any necessary adjustments on the study questionnaire depending on how well the respondents understood and gave responses.

### Data collection

Standardized structured quantitative interviewer-administered questionnaires were used, covering sociodemographic details, risky sexual behaviours, and HIV testing service utilization. Sociodemographic factors (age, sex, education level, marital status, average monthly income, religion) and indicators of risky sexual behaviours were assessed. Risky behaviours included sexual activity, age at first encounter, condom use, pregnancy prevention methods, transactional sex, multiple sexual partners, sex under the influence, and STI diagnosis in the past 12 months. HIV testing history was also assessed. The questionnaire was adapted with modification from 15, 19. Before the actual data collection, the questionnaire was pre-tested to see if participants are able to comprehend the questions, test the time taken. This was done in the indicated sub county which has similar characteristics with the study area. Data were collected between June and July 2023.

### Data analysis

Completed questionnaires were cross-checked for accuracy and completeness, then analysed using SPSS version 25. Descriptive statistics on sociodemographic characteristics such as age, sex, education level, marital status, average monthly income, religion, and risky sexual behaviours included sexual activity, age at first encounter, condom use, pregnancy prevention methods, transactional sex, multiple sexual partners, sex under the influence, and STI diagnosis in the past 12 months. Inferential statistics were employed. Regression analyses were performed to assess the association between the explanatory variables and each variable. Variables with a univariable p-value < 0.1 were included in multivariate logistic regression. Adjusted odds ratios, 95% confidence intervals, and p-values were reported, with significance set at p < 0.05.

### Ethical considerations

Ethical clearance was obtained from Jomo Kenyatta University of Agriculture and Technology Research Committee. Research permits were secured from the National Commission for Science Technology and Innovations (NACOSTI), and Nairobi County Ethical and Research Committee. Informed consent and assent were obtained from participants, ensuring full disclosure of study purpose, risks, and benefits. Strict confidentiality measures, including identity code usage and password protection, were implemented. Access to data was restricted, and anonymity was maintained during data sharing.

## Results

### Sociodemographic characteristics

The majority of the AGYW respondents (80.7%) were single, with the rest either married (18.2%) or separated (1.1%). Almost two-thirds (64.6%) were educated to secondary school level, while tertiary and primary levels accounted for 23.5% and 11.6% respectively ( Table 1) ^20^.

**Table 1.** To determine risky sexual behaviors associated with HIV testing among AGYW aged between 15-24 years in Kibra Sub County.

	Ever tested for HIV?		
Variable			
**Ever had sex?**	**Yes** **(N=230)**	**No** **(N=149)**	**Overall** **(N=379)**
No	13 (5.7%)	98 (65.8%)	111 (29.3%)
Yes	217 (94.3%)	51 (34.2%)	268 (70.7%)
**Age at first sexual intercourse (in ** **years)**	**Yes (N=217)**	**No (N=51)**	**Overall (N=268)**
Mean (SD)	17.9 (1.86)	17.3 (1.97)	17.8 (1.89)
Median [Min, Max]	18.0 [14.0, 24.0]	18.0 [13.0, 21.0]	18.0 [13.0, 24.0]
**Cash in return for sex?**	**Yes (N=217)**	**No (N=51)**	**Overall (N=268)**
No	175 (80.6%)	45 (88.2%)	220 (82.1%)
Yes	42 (19.4%)	6 (11.8%)	48 (17.9%)
**Have more than 1 lifetime sexual ** **partners?**	**Yes (N=217)**	**No (N=51)**	**Overall (N=268)**
No	174 (80.2%)	42 (82.4%)	216 (80.6%)
Yes	43 (19.8%)	9 (17.6%)	52 (19.4%)
**If yes to question 21 above, how ** **many?**	**Yes (N=43)**	**No (N=9)**	**Overall (N=52)**
Mean (SD)	2.67 (1.11)	2.33 (0.500)	2.62 (1.03)
Median [Min, Max]	2.00 [2.00, 7.00]	2.00 [2.00, 3.00]	2.00 [2.00, 7.00]
**Had more than 1 sexual partners** ** during the past 3 months?**	**Yes (N=217)**	**No (N=51)**	**Overall (N=268)**
No	185 (85.3%)	44 (86.3%)	229 (85.4%)
Yes	32 (14.7%)	7 (13.7%)	39 (14.6%)
**If yes to question 23 above, how** ** many?**	**Yes (N=32)**	**No (N=7)**	**Overall (N=39)**
Mean (SD)	2.37 (0.718)	2.29 (0.488)	2.35 (0.676)
Median [Min, Max]	2.00 [2.00, 5.00]	2.00 [2.00, 3.00]	2.00 [2.00, 5.00]
Missing	2 (6.3%)	0 (0%)	2 (5.1%)
**Did you use condom in your last ** **sexual intercourse?**	**Yes (N=217)**	**No (N=51)**	**Overall (N=268)**
No	156 (71.9%)	20 (39.2%)	176 (65.7%)
Yes	61 (28.1%)	31 (60.8%)	92 (34.3%)
**Did you use any method for** ** prevention of pregnancy during ** **your last sexual intercourse?**	**Yes (N=217)**	**No (N=51)**	**Overall (N=268)**
No	109 (50.2%)	20 (39.2%)	129 (48.1%)
Yes	108 (49.8%)	31 (60.8%)	139 (51.9%)
**Had sexual intercourse under the ** **influence of alcohol?**	**Yes (N=217)**	**No (N=51)**	**Overall (N=268)**
No	178 (82.0%)	47 (92.2%)	225 (84.0%)
Yes	39 (18.0%)	4 (7.8%)	43 (16.0%)
**Ever experienced gender-based** ** violence?**	**Yes (N=217)**	**No (N=51)**	**Overall (N=268)**
No	185 (85.3%)	47 (92.2%)	232 (86.6%)
Yes	32 (14.7%)	4 (7.8%)	36 (13.4%)
**Had any STI in the last 12 ** **months?**	**Yes (N=217)**	**No (N=51)**	**Overall (N=268)**
No	185 (85.3%)	44 (86.3%)	229 (85.4%)
Yes	32 (14.7%)	7 (13.7%)	39 (14.6%)

### Testing services utilization

Regarding HIV testing, participants from Sarangombe had the highest testing rate 47.4% (179), followed by Lindi 28.7% (111) and Laini Saba 23.9% (89). 60.7% (230) of the AGYW had tested for HIV, with a majority 71.3% (164) being in the 20–24 age group and 28.7% (66) being among participants aged 15–19. Education played a role in utilization of HIV testing services as 63.5% (146) of those with secondary education and 23.3% (56) with tertiary education having been tested, compared to only 12.2% (28) with primary education. Employment status influenced testing, with most unemployed participants, 79.6% (183), having tested for HIV, while business, and employed individuals had lower and similar testing rates at 10.4% (24), and 10.0% (23) respectively. Participants earning less than 10,000 monthly had a testing rate of 64.8% (149), contrasting sharply with the 1.3% (3) testing rate among those earning between 20,000 and 30,000. Marital status showed differences, as 69.6% (160) of single participants were tested compared to 28.7% (66) married participants. In terms of religion, 84.8% (195) of Christians and 15.2% (35) of Muslims underwent HIV testing, the overall testing prevalence was 60.7%, as seen in Table 1.

### Bivariate analysis

Adolescent girls and young women aged 20–24 years exhibited a substantial likelihood of testing (OR: 4.50, 95% CI: 2.91, 7.04, p < 0.001), while not employed participants were markedly less likely to get tested (OR: 0.23, 95% CI: 0.04, 0.50, p < 0.004). Married individuals exhibited a significantly higher likelihood of HIV testing (OR: 20.1, 95% CI: 7.26, 83.32, p < 0.001). No significant association was found between religion and utilization of HIV testing services. For AGYW who had ever engaged in sexual activity, the likelihood of testing was notably elevated (OR: 32.08, 95% CI: 17.22, 64.20, p < 0.001). Furthermore, individuals not using condoms in their last sexual encounter demonstrated a higher probability of HIV testing (OR: 3.96, 95% CI: 2.12, 7.58, p < 0.001). Experiencing gender-based violence significantly increased the likelihood of testing (OR: 4.65, 95% CI: 1.93, 13.87, p = 0.002), and participants reporting recent STI contraction were also more inclined to undergo HIV testing (OR: 2.85, 95% CI: 1.33, 6.81, p = 0.011), as detailed in Table 2.

**Table 2.** Univariable logistic regression model assessing sociodemographic and risky sexual behaviour factors associated with utilization of HIV testing services.

Variable	Category	Crude OR	95% CI	P-value
Ward	Laini Saba	Ref		
	Lindi	0.91	0.51, 1.60	0.737
	Sarangombe	0.96	0.57, 1.62	0.886
Age (ungrouped)		1.61	1.44, 1.82	<0.001*
Age (grouped)	15–19 years	Ref		
	20–24 years	4.5	2.91, 7.04	<0.001*
Education level	No formal education	Ref		
	Primary	-	-	0.979
	Secondary	-	-	0.979
	Tertiary	-	-	0.979
Employment status	Business	Ref		
	Not employed	0.23	0.04, 0.50	0.004365*
	Employed	0.26	0.05, 0.96	0.060
Average monthly income	No income	0.23	0.01, 1.86	0.210
	<10,000	0.72	0.04, 5.74	0.778
	10,000 - 20,000	2.17	0.09, 22.71	0.544
	20,000 - 30,000	Ref		
Marital status	Single	Ref		
	Married	20.1	7.26, 83.32	<0.001*
	Separated	-	-	0.983
Religion	Christian	1.02	0.56, 1.79	0.954
	Muslim	Ref		
Ever had sex	No	Ref		
	Yes	32.08	17.22, 64.20	<0.001*
Age at first sex (in years)		1.18	1.00, 1.40	0.053
Cash in return for sex	No	Ref		
	Yes	1.8	0.77, 4.95	0.208
1+ lifetime sex partners	No	Ref		
	Yes	1.15	0.54, 2.69	0.725
Used condom last sex	No	3.96	2.12, 7.58	<0.001*
	Yes	Ref		
Contraceptive last sex	No	1.49	0.81, 2.78	0.204
	Yes	Ref		
Sex under alcohol influence	No	Ref		
	Yes	2.63	0.996, 9.08	0.079
Experienced GBV	No	Ref		
	Yes	4.65	1.93, 13.87	0.002*
STI last 12 months	No	Ref		
	Yes	2.85	1.33, 6.81	0.011*

### Multivariable analysis

AGYW aged 20–24 years were 2.40 times more likely to undergo HIV testing (AOR: 2.40, 95% CI: 1.44, 4.02, p = 0.001). Additionally, married AGYW exhibited a significantly higher likelihood of getting tested, with an odds ratio of 10.50 (95% CI: 3.63, 44.58, p = 0.000). Among AGYW aged 15–24 years, those who did not use condoms in their last sexual encounter were 4.27 times more likely to opt for HIV testing (AOR: 4.27, 95% CI: 2.25, 8.30, p < 0.001). These findings highlight the substantial impact of age, marital status, and condom usage on HIV testing behaviours within this demographic group ( Table 3).

**Table 3.** Multivariable logistic regression model assessing sociodemographic and risky sexual behaviour factors associated with utilization of HIV testing services.

	Variable	Adjusted OR	95% CI	P-value
Age (grouped)	15–19 yrs.	Ref		
	20–24 yrs.	2.4	1.44, 4.02	<0.001*
Employment status	Business	Ref		
	Not employed	0.81	0.16, 3.15	0.780
	Employed	0.52	0.10, 2.25	0.398
Average monthly income	No income	0.42	0.02, 5.12	0.518
	<10,000	0.64	0.03, 7.18	0.734
	10,000 - 20,000	1.22	0.05, 15.76	0.883
	20,000 - 30,000	Ref		
Marital status	Single	Ref		
	Married	10.5	3.63, 44.58	<0.001*
	Separated	-	-	0.984
Used condom last sex	No	4.27	2.25, 8.30	<0.001*
	Yes	Ref		
Sex under alcohol influence	No	Ref		
	Yes	2.99	1.04, 11.02	0.064
Experienced GBV	No	Ref		
	Yes	1.8	0.58, 6.88	0.341
STI last 12 months	No	Ref		
	Yes	0.66	0.25, 1.89	0.411

## Discussion

The study findings indicated that adolescent girls and young women between the age of 20–24 years had testing rate of 71.3% or were 4.5 times likely to test for HIV. The findings concur with studies conducted in different countries which attributed age among other sociodemographic factors to have a significant association with utilization of HIV testing services among the adolescent girls and young women as those aged 20 and above were more likely to utilize HIV testing services compared to those aged between 15–19 years in most Sub-Saharan Africa ^1, 21^. Better testing rates for AGYW between 20–24 years may imply that these are young women who have completed secondary education and have a higher chance of getting or accessing information on HIV awareness, better understating on the importance of HIV testing which may drive them to make evidence-based decision thus increasing their chances of being tested. Additionally, this group is more likely to have sexual partners older than them due to material benefits to meet other basic needs that they may not be getting from their parents or guardians, further, they may not be first-time testers but had been tested for HIV even before the study ^16^. On the contrast, there is probability that AGYW younger than 20 years of age have shorter sexual experience and hence less informed on sexual matters compared to older one thus disparity in HIV testing.

Participants who had attained secondary and tertiary education had better testing rates at 63.5% and 23.5% respectively an indication that the more the AGYW are educated, the more likely they are to test for HIV compared to less educated, this is also true since the only participant who did not have formal education had not tested for HIV. Other studies indicate that people who are educated up to secondary education level and above are more likely to utilize HIV testing services compared to those with primary or no education ^22, 23^. With majority having attained secondary education, it points to better overall achievement in school enrolment, greater empowerment, and knowledge on sexual and reproductive health among the residents and a pointer to association between education level and utilization of HIV testing.

The study findings indicate that the testing prevalence for the participants in Kibra which is an informal settlement was 60.7%, the finding are not consistent with a study in Viwandani and Korogocho slums in Nairobi which showed that AGYW are less aware of their HIV status compared to those above 25 years ^16^. Improvement in testing rates among this group may be attributed to the concerted efforts by the Ministry of Health in implementing various interventions like campaigns promoting HIV testing, increased availability of HIV testing centres and youth friendly HIV testing services among all geared towards ending HIV pandemic and achieving UNAIDS target by 2030.

Majority of the participants who were married had tested for HIV which may be attributed to the program of prevention of perinatal transmission which requires that all pregnant and expectant mothers attending antennal clinics should be initiated on provider HIV testing services for them to understand the potential of reducing the chances of transmitting HIV to their unborn child hence are more likely to be tested for HIV. Studies in sub-Saharan Africa have shown that most indicated that married people were more likely to utilize HIV testing services compared to those not married or in any union as more countries adopt policies to encourage HIV testing ^8, 24, 25^. This implies the importance of prevention of mother to child transmission (PMTCT) program in sensitizing expectant mothers on the advantages of undergoing HIV testing during pregnancy, labour, and delivery and postnatally, the importance of training health care workers and care givers on optimizing the interventions of HIV prevention including awareness on HIV and differentiated prevention services like PrEP that focuses on AGYW.

The study demonstrated that AGYWs who had sexual intercourse without using condom, who had sexual intercourse under the influence of alcohol, who had contracted STI in the last 12 months and who had experienced gender-based violence were more likely to test for HIV. Similarly, studies in Zimbabwe and Ethiopia indicated that AGYW who engage in sex for monetary gains are less likely to utilize HIV testing services compared to those who don’t do it for money, those who have multiple sexual partners are likely to test compared to those with only one, additionally, those who use condoms during sexual intercourse are less likely to test compared to those who don’t use ^8, 9^. The findings of the study underscore the need for interventions targeting the young AGYWs to understand the risks involved in early sexual debut and other available protective measures to prevent HIV transmission such as Pre and Post exposure prophylaxis.

## Study limitations

The study was cross sectional, no causal conclusions or inference on causality can be drawn from the assessed factors, although the sample included in the study was representative, Kibra is only one of seventeen (17) sub counties in Nairobi County, it’s an informal settlement hence study findings may not be generalizable to other formal and urban settlements. The study relied on self-report on the variables assessed which may be influenced by social disparities, several other variables affecting utilization of HIV testing services such as individual level factors, hospital level factors, perceived vulnerabilities and risks, access to testing sites were not assessed hence should be included in future studies. However, the study provided pivotal insights necessary for developing targeted behavioural interventions that address the specific challenges faced by AGYW, thus contributing significantly to the design of more effective public health strategies.

## Conclusion

Risky sexual behaviours among the AGYW in Kibra sub county was noted to be common and may mirror other findings on HIV burden among populations in informal settlements. This study established a clear link between sociodemographic characteristics and risky sexual behaviours and HIV testing among AGYW, with a 60.7% testing prevalence against a target of 95%, efforts to enhance testing rates are vital. Interventions should align with acceptable methods, focusing on this affected population to ensure effective HIV care and prevention. Social support and protective measures for AGYW is crucial in HIV response. Despite limitations, the study contributes to knowledge on the association between risky sexual behaviours and HIV testing in Kenya, more research should be conducted considering other factors such as individual level factors, hospital level factors, perceived vulnerabilities and risks, access to testing sites among other variables.

## What is already known on this topic

1. **Disparities in HIV testing rates:** Existing research has shown disparities in HIV testing rates among different demographic groups, particularly among adolescent girls and young women (AGYW). These disparities are often influenced by factors such as age, education, and socioeconomic status.
2. **Impact of risky sexual behaviours:** Previous studies have established a link between risky sexual behaviours and HIV testing patterns. Risky behaviours, including sex without the use of prevention tools and multiple sexual partners, have been identified as significant factors influencing HIV testing behaviour among AGYW.

## What this study adds

1. **Localized insights into testing disparities:** This study provides localized insights into HIV testing disparities within specific communities, shedding light on differences in testing rates among various wards. Understanding these community-specific variations is essential for tailoring targeted interventions and healthcare resources effectively.
2. **Comprehensive analysis of sociodemographic factors:** Unlike some previous studies, this research comprehensively examines a range of sociodemographic factors, including education, employment status, income, and marital status, to identify nuanced influences on HIV testing among AGYW. By analysing these multifaceted factors, this study enriches the understanding of the complex interplay between sociodemographic variables and HIV testing behaviour in this vulnerable population.
3. **Behavioural contextualization of HIV testing:** This study goes beyond numerical analysis by delving into the behavioural context surrounding HIV testing among AGYW. By exploring risky sexual behaviours in detail, such as transactional sex, alcohol-induced encounters, and STI diagnoses, this research offers a nuanced understanding of the psychosocial factors influencing HIV testing decisions. These insights are pivotal for developing targeted behavioural interventions that address the specific challenges faced by AGYW, thus contributing significantly to the design of more effective public health strategies.

## Ethics and consent

Ethical clearance was obtained from Jomo Kenyatta University of Agriculture and Technology Research Committee (ref number. JKU/ISERC/02316/0832; 23 February 2023). Research permits were secured from the National Commission for Science Technology and Innovations (NACOSTI) and Nairobi County Ethical and Research Committee. Written informed consent was obtained from participants (or the guardians/parents of the participants under 18 years old, with assent being obtained from the participants) before they filled out the questionnaire, ensuring full disclosure of study purpose, risks, and benefits.

## Data Availability

Dryad: Risky sexual behaviours and utilization of HIV testing services among the adolescent girls and young women aged between 15–24 years in Kibra Sub County, Nairobi County, Kenya. https://doi.org/10.5061/dryad.kh18932fs ^20^. Data are available under the terms of the Creative Commons Zero "No rights reserved" data waiver (CC0 1.0 Public domain dedication).
