# Supplementary material for: Risky sexual behaviours and utilization of HIV testing services among the adolescent girls and young women aged between 15-24 years in Kibra Sub County, Nairobi County, Kenya
Source: Open Res Eur. 2025 Jan 31;4:106. Originally published 2024 May 29. [Version 2] doi: 10.12688/openreseurope.17609.2 (PMC11847150; doi:10.12688/openreseurope.17609.2)
Supplement: Peer review history

# Reviewer report: Reviewer response for version 2

**DOI:** 10.21956/openreseurope.21104.r50555

**Published:** 2025-02-22

**Authors:** Alfred Musekiwa

**Affiliations:** 1 University of Pretoria, Pretoria, Gauteng, South Africa

All my comments have been addressed. I have no further comments to make.

Is the study design appropriate and does the work have academic merit?

Yes

Is the work clearly and accurately presented and does it cite the current literature?

Yes

If applicable, is the statistical analysis and its interpretation appropriate?

Partly

Are all the source data underlying the results available to ensure full reproducibility?

Yes

Are the conclusions drawn adequately supported by the results?

Yes

Are sufficient details of methods and analysis provided to allow replication by others?

Partly

Reviewer Expertise:

Biostatistics, HIV/AIDS among adolescent girls and young women in Sub-Saharan Africa

I confirm that I have read this submission and believe that I have an appropriate level of expertise to confirm that it is of an acceptable scientific standard.

# Reviewer report: Reviewer response for version 2

**DOI:** 10.21956/openreseurope.21104.r50554

**Published:** 2025-02-06

**Authors:** Elizabeth Kiilu

**Affiliations:** 1 University of Bradford, Bradford, UK

The article has addressed my comments satisfactorily.

Is the study design appropriate and does the work have academic merit?

Yes

Is the work clearly and accurately presented and does it cite the current literature?

Yes

If applicable, is the statistical analysis and its interpretation appropriate?

Partly

Are all the source data underlying the results available to ensure full reproducibility?

Yes

Are the conclusions drawn adequately supported by the results?

Partly

Are sufficient details of methods and analysis provided to allow replication by others?

Partly

Reviewer Expertise:

Public Health and health systems management

I confirm that I have read this submission and believe that I have an appropriate level of expertise to confirm that it is of an acceptable scientific standard.

# Reviewer report: Reviewer response for version 1

**DOI:** 10.21956/openreseurope.19028.r48451

**Published:** 2025-01-13

**Authors:** Elizabeth Kiilu

**Affiliations:** 1 University of Bradford, Bradford, UK

Risky sexual behaviours and utilization of HIV testing services among the adolescent girls and young women aged between 15-24 years in Kibra Sub County, Nairobi County, Kenya  

**Abstract:** 

 -The results in the abstract are not well presented with the respective Confidence intervals. All statistics must be fully presented.  

 - Was ethical approval obtained? Needs to be reflected in the methods section of the abstract. 

 Conclusions and recommendations should match what was presented as the main findings. You talk about age, secondary education and those married as being more likely to be tested in reference to which specific categories? i.e. what are the reference points of these findings so that it's clearer? Other findings highlighted are condom use, GBV and STIs...These findings need to be matched with your conclusions and recommendations.

 You have provided several findings with only one conclusion on risky sexual behaviours. This is a positive finding as those who are engaging in risky sexual behaviours are actually more likely to seek HIV services. This is a good thing! It's exactly what needs reinstated among teenagers engaging in risky sexual behaviours. A more Profound finding is the low testing prevalence...what can be done about this?

 What are the outcomes of those with low testing rates?

 Are younger people having lower testing rates?

 What can be done to help?

 Are those with lower levels of education less likely to seek testing services and what can be done to improve this?

 The results need to be presented in a clearer manner to show the implication of the said findings using the respective reference groups.

 Essentially in the abstract, you present the main findings of the study (1-3) then conclude on these findings and give recommendations based on these main findings. You don't need to present all the positive findings in the abstract.

**Introduction:**

 This section is fairly well written with excellent justification of why AGYW alongside current epidemiological statistics. Some areas that could use some improvement in this section: 

 The paragraph below could use more elaboration for some of the indicators. For instance, age is a significant finding in your study...elaborate further how this may or may not be a factor in other studies and the implications of age... what about stigma? Explore this further as well as attitudes and implications of early sexual debut. These are all drivers or barriers for testing and could be explored in more detail.  

 “Additionally, studies across different parts of the globe have positively associated risky sexual behaviours such as age of respondents, early sexual debut, stigma, attitudes with predisposing AGYW to HIV and utilization of HIV testing services 10– 12 

**Methods:**

 -Cite the source of the demographics in the first paragraph. 

 -In the abstract you say that you used logistic regression, yet your Dependent variable in the methods is stated as binary outcome. So, did you use logistic regression, or you used binary regression? 

 -In the exclusion criteria you cannot exclude those who were not present at in the HH as they are actually not there anyway. Remove that statement.  

 -What was the power of the study? 

 -Include the ethical approval number from Jomo Kenyatta University of Agriculture and Technology Research Committee.   

**Results:**

- Table 1 results (descriptive statistics) are clear. 

- Table 2 results (not clear what the authors are testing.) Are these for test of association i.e Chi square test? If this is the case you have wrongly presented these results as you don't have OR in chi square tests. Additionally, some data is missing in the table i.e. level of education data is missing yet a p-value is indicated. If this table is not testing associations using Chi square test as indicated in your methods, you need to include your test of association table. 

-  If you are going to present crude ORs you should give a statement of caution that these do not take care of possible confounders, and the results should be interpreted as such.  

- Multivariable analysis: when presenting your results, it's important to indicate the reference group (which shows what category of independent variables you are comparing to) for instance in the statement below: 

AGYW aged 20–24 years were 2.40 times more likely to undergo HIV testing (AOR: 2.40, 95% CI: 1.44, 4.02, p = 0.001).  

 The above information does not mean much if you do not give the reader what you are making reference to. This could have been stated as follows: 

 AGYW aged 20–24 years were 2.40 times more likely to undergo HIV testing (AOR: 2.40, 95% CI: 1.44, 4.02, p = 0.001) relative to their younger counterparts aged 15-19 years (95%CI................). This should be reflected in all the results you present even in the discussion section. 

**Discussion:**

 -The first paragraph and subsequent ones giving results should indicate the reference category. I.e. The study findings indicated that adolescent girls and young women between the age of 20–24 years had testing rate of 71.3% or were 4.5 times likely to test for HIV compared to their younger counterparts/the younger adolescents aged 15-19 years. This has already been explained previously.  

 - In the below paragraph what was the testing rate at Korogocho/viwandani compared to KIbra? You only mention it was less...and what could be the possible explanation for this disparity given that they are both informal settlements? 

 “The study findings indicate that the testing prevalence for the participants in Kibra which is an informal settlement was 60.7%, the finding are not consistent with a study in Viwandani and Korogocho slums in Nairobi which showed that AGYW are less aware of their HIV status compared to those above 25 years 16.” 

**Conclusions:**

 The conclusions need to be matched with the findings of the study. This has been alluded to in the abstract section on how to go about it.

Is the study design appropriate and does the work have academic merit?

Yes

Is the work clearly and accurately presented and does it cite the current literature?

Yes

If applicable, is the statistical analysis and its interpretation appropriate?

Partly

Are all the source data underlying the results available to ensure full reproducibility?

Yes

Are the conclusions drawn adequately supported by the results?

Partly

Are sufficient details of methods and analysis provided to allow replication by others?

Partly

Reviewer Expertise:

Public Health and health systems management

I confirm that I have read this submission and believe that I have an appropriate level of expertise to confirm that it is of an acceptable scientific standard, however I have significant reservations, as outlined above.

# Reviewer report: Reviewer response for version 1

**DOI:** 10.21956/openreseurope.19028.r43262

**Published:** 2024-09-02

**Authors:** Alfred Musekiwa

**Affiliations:** 1 University of Pretoria, Pretoria, Gauteng, South Africa

Summary of article - The authors determined prevalence and determinants of HIV testing among AGYW in a sub-county in Kenya. Sufficient and up-to-date literature on the field has been cited, however the authors can include aspects of HIV self-testing among the study population, and also contextualise the study according to SDG's and Kenya's Ministry of Health priorities. Although appropriate statistical methods were used, such as logistic regression, the authors were not transparent enough to show sample size calculation parameters or assumptions. Importantly, the authors did not adjust their analyses for survey sampling. The findings have been properly presented, however the interpretation of odds ratios, in some instances, were incorrect. The Discussions and conclusions are congruent with the findings.

 Below are the detailed comments, section by section;

 ABSTRACT

 Methods

 1) Specify multivariable logistic regression model will be used to determine factors associated with HIV testing uptake

 Results

 The interpretation needs to be improved. The odds ratios should be interpreted in terms of the odds are x times higher in this group compared to that group. The interpretation in the abstract and manuscript is for risk ratios and not odds ratios.

 Conclusions

 The recommendations can be made more specific.

 INTRODUCTION

 Paragraph 3 - The language needs to be improved, for example do not use the word "don't". Also last sentence, age is not a risky sexual behaviour.

 Paragraph 4, specify the years for these prevalences for the different countries.

 Last paragraph, the authors may want to specify the risky sexual factors that the study analysed.

 General comment: The authors may want to link the study with sustainable development goals (SDG's) and also the priorities of Kenya MOH.

 METHODS

 Study site and population:

 The "multistage sampling technique" used has an implication on the analysis method - it requires the authors to adjust the analysis for complex survey sampling using survey weights.

 Sample size determination:

 More details are required on sample size calculation to enable a statistician to check if the calculation is correct.

 Data analysis:

 The analysis should have been adjusted for unequal sampling probabilities due to the complex survey design.

 RESULTS

 Testing service utilization: give units for the currency reported.

 Bivariate analysis:

 Table 2, it may benefit to add a column showing percent with outcome - to show these percent's for each category.

 Multivariate analysis:

 The interpretation "AGYW aged 20–24 years were 2.40 times more likely to undergo HIV testing (AOR: 2.40, 95% CI: 1.44, 4.02, p = 0.001)." is wrong, since the authors are interpreting an odds ratio as if it was a risk ratio; the authors can use the language of saying "The odds of HIV testing were 2.4 times higher...".

 The comment above applies to the rest of the paragraph.

 Also the authors need to indicate the total number in the final multivariable logistic regression model.

 DISCUSSION

 First sentence, see comment above regarding interpretation of odds ratios.

 The authors may want to discuss how HIV self-testing can be promoted to increase uptake of HIV testing services among AGYW in Kenya.

 When discussing marriage, the authors can also bring out how pre-marital HIV testing can also be used to promote HIV testing.

 CONCLUSIONS

 The statement "60.7% testing prevalence against a target of 95%, efforts to enhance testing rates are vital" is incorrect because the denominator for the first-95 of 95-95-95 is people living with HIV and not the general population like in this case. I suggest that the authors simply state that the percentage is low.

Is the study design appropriate and does the work have academic merit?

Yes

Is the work clearly and accurately presented and does it cite the current literature?

Yes

If applicable, is the statistical analysis and its interpretation appropriate?

Partly

Are all the source data underlying the results available to ensure full reproducibility?

Yes

Are the conclusions drawn adequately supported by the results?

Yes

Are sufficient details of methods and analysis provided to allow replication by others?

Partly

Reviewer Expertise:

Biostatistics, HIV/AIDS among adolescent girls and young women in Sub-Saharan Africa

I confirm that I have read this submission and believe that I have an appropriate level of expertise to confirm that it is of an acceptable scientific standard, however I have significant reservations, as outlined above.

# Response

**Published:** 2024-09-10

**Authors:** Onesmus Mutie

**Affiliations:** Environmental Health and Disease Control, Jomo Kenyatta University of Agriculture and Technology, Nairobi, Nairobi County, Kenya

**Alfred Musekiwa**, University of Pretoria, Pretoria, Gauteng, South Africa Reviewer

 Please take a look of the updated manuscript with tracked changes based on reviewers comments - https://s3-eu-west-1.amazonaws.com/openreseurope/linked/237947.Published_article_Reviewer_comments_addressed__onesmus_mutie.docx​​​​​​​  (17609 - Revised Manuscript as per Reviewer Alfred Musekiwa comments)

**Summary of article - The authors determined prevalence and determinants of HIV testing among AGYW in a sub-county in Kenya. Sufficient and up-to-date literature on the field has been cited, however the authors can include aspects of HIV self-testing among the study population, and also contextualise the study according to SDG's and Kenya's Ministry of Health priorities. Although appropriate statistical methods were used, such as logistic regression, the authors were not transparent enough to show sample size calculation parameters or assumptions. Importantly, the authors did not adjust their analyses for survey sampling. The findings have been properly presented, however the interpretation of odds ratios, in some instances, were incorrect. The Discussions and conclusions are congruent with the findings.**

**Below are the detailed comments, section by section;**

**ABSTRACT**

**Methods**

**1) Specify multivariable logistic regression model will be used to determine factors associated with HIV testing uptake**

**a manual stepwise procedure was used to enter variables with p-values < 0.1 in the bivariate model analysis into the multivariate logistic regression model**

**Results**

**The interpretation needs to be improved. The odds ratios should be interpreted in terms of the odds are x times higher in this group compared to that group. The interpretation in the abstract and manuscript is for risk ratios and not odds ratios.**

**Response**

 The odds of undergoing HIV testing was higher for AGYW aged 20-24 years (aOR: 2.40, 95% CI: 1.44, 4.02, p = 0.001). Additionally, married AGYW exhibited significantly higher odds of getting tested, (aOR 11.04, 95% CI: 3.84, 46.75, p = 0.001). AGYW who did not use condoms in their last sexual encounter had a higher odds of opting for HIV testing (aOR: 4.27, 95% CI: 2.25, 8.30, p < 0.001). These findings highlight the substantial impact of age, marital status, and condom usage on HIV testing behaviors within this demographic group

**Conclusions**

**The recommendations can be made more specific.**

**Response**

 Interventions should align with acceptable methods, focusing on this high-risk group to ensure effective HIV care and prevention. Consider integration of HIV services in various service delivery points, upscale the uptake of HIV self-testing for both oral and blood based, social support and protective measures for AGYW is crucial in HIV response.

**INTRODUCTION**

**Paragraph 3 - The language needs to be improved, for example do not use the word "don't". Also last sentence, age is not a risky sexual behaviour.**

**Response**

 The word don’t has been reworded

 Age removed as risky behavior since it is covered in the early sexual debut

**Paragraph 4, specify the years for these prevalences for the different countries.**

**Years indicated**

**Last paragraph, the authors may want to specify the risky sexual factors that the study analysed.**

**Response**

 risky sexual behaviours such as early sexual debut, condom use, substance use, having multiple sexual partners, transactional sex, among others

**General comment: The authors may want to link the study with sustainable development goals (SDG's) and also the priorities of Kenya MOH.**

**Response**

 The study will contribute to the body of knowledge regarding HIV testing services, add to the efforts to decrease HIV infections among the AGYW, contribute to interventions geared towards attainment of the 95-95-95 strategy to end HIV epidemic by 2030 and contribute towards attainment of SDG 3 target 3.3 which aims to fight communicable diseases.

**METHODS**

**Study site and population:**

**The "multistage sampling technique" used has an implication on the analysis method - it requires the authors to adjust the analysis for complex survey sampling using survey weights.**

**Response:**

 The study used a four multistage sampling technique;

1. Using random numbers, 3 wards from the 5 were selected (Sarang’ombe, Laini saba and Lindi)
2. According to 2019 Census, the population of AGYW in Sarang’ombe ward is 5,741, Laini saba 2,870 and Lindi 3,565. This population is projected to grow at a rate of 2.2% per year which means that the current population will be (Sarang’ombe 6,263, Laini saba 3,131 and Lindi 3,889 giving a total population for the three wards as 13,283.
3. Proportions were used to allocate sample for each ward i.e Sarang’ombe ward 47% of 379 (179), Laini saba 24% of 379 (89) and Lindi 29% of 379 (111).
4. The study utilized the random walk sampling method whereby in each of the selected ward, a common meeting place, either a market, hospital or school was chosen as the starting point, with the help of a local guide, one route from the selected meeting point was chosen at random and the data collection proceeded along that route until the minimum sample size was reached. The first household along the route was skipped and the next household was assessed for presence of the population of interest. The data collector continued to skip one household in this manner until the minimum sample size was achieved. For households with more than one AGYW, use of random numbers written yes and no was used so that the one that picks yes was interviewed. Households without AGYW were skipped.

**Sample size determination:**

**More details are required on sample size calculation to enable a statistician to check if the calculation is correct.**

**Response**

 Sample size was calculated using Cochran formula (1977) since the estimated population of AGYW in Kibra Sub County is above 10,000



 Where;

**n _0_** - is the sample size;

**Z ^2 = ^** Is the standard normal deviate for alpha at 95% confidence level (1.96); 

**E = **is the desired level of precision (0.05);

**P = **56% proportion of AGYW ever tested for HIV and know their status in Kenya (Kenya Demographic Health Survey (KDHS 2022);

**Q = **is 1-p. (0.44);

 The value for Z is found in statistical tables which contain the area under the normal curve. i.e.  Z = 1.96 for 95 % level of confidence

              = 378.6 ~ (379)

**Data analysis:**

**The analysis should have been adjusted for unequal sampling probabilities due to the complex survey design.**

**Because the data collection tool was interviewer administered, there were no chances of non-response, this meant that there was no need of adjustment to cater for non-response.**

**RESULTS**

**Testing service utilization: give units for the currency reported.**

**Bivariate analysis:**

**Table 2, it may benefit to add a column showing percent with outcome - to show these percent's for each category.**

**Percentage added in the table**

**Multivariate analysis:**

**The interpretation "AGYW aged 20–24 years were 2.40 times more likely to undergo HIV testing (AOR: 2.40, 95% CI: 1.44, 4.02, p = 0.001)." is wrong, since the authors are interpreting an odds ratio as if it was a risk ratio; the authors can use the language of saying "The odds of HIV testing were 2.4 times higher...".**

**The comment above applies to the rest of the paragraph.**

**Also the authors need to indicate the total number in the final multivariable logistic regression model.**

**Response**

 Adjustment done to reflect suggested comments

**DISCUSSION**

**First sentence, see comment above regarding interpretation of odds ratios.**

**The authors may want to discuss how HIV self-testing can be promoted to increase uptake of HIV testing services among AGYW in Kenya.**

**When discussing marriage, the authors can also bring out how pre-marital HIV testing can also be used to promote HIV testing.**

**CONCLUSIONS**

**The statement "60.7% testing prevalence against a target of 95%, efforts to enhance testing rates are vital" is incorrect because the denominator for the first-95 of 95-95-95 is people living with HIV and not the general population like in this case. I suggest that the authors simply state that the percentage is low.**

**Response**

 Adjusted accordingly
